# Acupuncture alleviates hemorrhagic transformation after delayed rt-PA treatment for acute ischemic stroke by regulating the mitophagy-NLRP3 inflammasome pathway

**DOI:** 10.3389/fneur.2025.1533092

**Published:** 2025-04-07

**Authors:** Tao Jiang, Qianqian Liu, Huanhuan Liu, Zheng Huang, Mengning Yang, Peiyan Huang, Yiting Shen, Yangyang Song, Wentao Xu, Xinchang Zhang, Guangxia Ni

**Affiliations:** ^1^College of Acupuncture-Moxibustion and Tuina, Nanjing University of Chinese Medicine, Nanjing, China; ^2^Key Laboratory of Acupuncture and Medicine Research of Ministry of Education, Nanjing University of Chinese Medicine, Nanjing, China; ^3^College of Chinese Medicine, Nanjing University of Chinese Medicine, Nanjing, China

**Keywords:** acupuncture, mitophagy, NLRP3 inflammasome, hemorrhagic transformation, rt-PA thrombolysis, acute ischemic stroke

## Abstract

**Background:**

The clinical application of recombinant tissue plasminogen activator (rt-PA) is significantly constrained by hemorrhagic transformation (HT), a common and severe complication following thrombolysis for ischemic stroke. Notably, the mitochondrial injury-mediated NLRP3 inflammasome plays a crucial role in HT after delayed rt-PA thrombolysis in acute ischemic stroke. Although acupuncture has demonstrated antioxidant and anti-inflammatory effects in acute cerebral infarction, its impact on delayed rt-PA thrombolysis, especially concerning mitophagy and the NLRP3 inflammasome, remains unclear. This study investigates how acupuncture protects against HT resulting from mitochondrial damage and NLRP3 inflammasome activation after delayed rt-PA thrombolysis in acute cerebral stroke.

**Methods:**

We selected an embolic stroke model in rats and assessed brain injury after delayed rt-PA in acute ischemic stroke using neurological deficit score, volume of brain infarct, the permeability assay of the blood–brain barrier (BBB), and HT. Then, the levels of proteins and mRNA involved in mitophagy and the NLRP3 inflammasome pathway were measured by western blot and real-time PCR. The levels of interleukin-18 (IL-18) and interleukin-1β (IL-1β) were assessed using enzyme-linked immunosorbent assay (ELISA). Morphological changes in the BBB and mitochondria of neurons were observed via transmission electron microscopy.

**Results:**

Acupuncture significantly improved neurological deficit scores, volume of cerebral infarction, BBB destruction, and HT in an embolic stroke model rat. Furthermore, acupuncture induced mitophagy and substantially downregulated the activity of the NLRP3 inflammasome. Additionally, the use of mitochondrial inhibitors significantly reversed the suppressive impact of acupuncture on the NLRP3 inflammasome.

**Conclusion:**

Acupuncture can promote mitophagy and suppress NLRP3 inflammasome activation to decrease HT after delayed rt-PA therapy for acute ischemic stroke.

## 1 Introduction

Stroke is a leading cause of disability and death in China, with ischemic stroke being the most common type, representing approximately 86.8% of all cases (1). Currently, the best treatment for ischemic stroke is intravenous recombinant tissue fibrinogen activator (rt-PA). Nevertheless, this approach is constrained by a short time window (within 4.5 h) (2). Delayed rt-PA thrombolysis may lead to several severe complications, including hemorrhagic transformation (HT), cerebral edema, and reperfusion injury, with HT being the most frequently observed complication (3, 4).

The inflammasome is strongly implicated in the development of ischemic stroke (5). The Nod-like receptor protein 3 (NLRP3) inflammasome is a complex multiprotein structure that includes the NLRP3 receptor, apoptosis-associated speck-like protein containing the caspase-recruiting domain (ASC), and the precursor form of Caspase-1 (Pro-caspase-1) (6). The activated NLRP3 complex eventually triggers the release of interleukin-18 (IL-18) and interleukin-1β (IL-1β). These cytokines trigger downstream signaling pathways and initiate inflammatory responses. This process disrupts tight junction (TJ) proteins, thereby enhancing the permeability of the blood–brain barrier (BBB) and increasing the incidence of HT (7, 8).

Mitophagy can selectively eliminate defective or impaired mitochondria and is a critical mechanism for maintaining the quality of mitochondria (9). Extensive research has demonstrated that the Pink1 (PTEN-inducible putative kinase 1)/Parkin (E3 ubiquitin ligase) pathway constitutes the canonical mitophagy mechanism (10, 11). Upon mitochondrial injury, Pink1 is activated and subsequently recruits Parkin to the outer membrane (12). Activated Parkin is identified by P62 (an autophagy junction protein) and facilitates the transport of mitochondria to autophagic vesicles through its interaction with the protein LC3, which induces the generation of mitochondrial autophagic vesicles that ultimately merge with lysosomes to eliminate damaged mitochondria (9). Meanwhile, some reports have suggested that mitophagy mediated by Pink1/Parkin is an essential mechanism for suppressing the activity of the NLRP3 inflammasome (13, 14). Moreover, mitophagy may mitigate brain damage in ischemic stroke rats via its inhibitory effect on NLRP3 inflammasome activation (15).

Accumulating evidence indicates that acupuncture may substantially decrease neurological impairment score and cerebral infarction volume in ischemic stroke rats while also inhibiting NLRP3 and Caspase-1 expression, thereby providing a neuroprotective effect (16). Meanwhile, acupuncture can protect neurons from damage through enhancing mitophagy through the Pink1/Parkin-dependent pathway (17).

Although acupuncture has been demonstrated to exert neuroprotective effects in cerebral ischemia, its effects on mitophagy and interactions with NLRP3 inflammasomes remain unknown. This study examined the potential of acupuncture to mitigate HT following delayed rt-PA thrombolysis in acute ischemic stroke rats by modulating the mitophagy-NLRP3 inflammasome pathway.

## 2 Materials and methods

### 2.1 Animals

Male Sprague–Dawley (SD) rats were sourced from Beijing Viton Lever (SCXK jing 2021–0006) with an average weight of 310 ± 20 g. The rats had unrestricted water and food access. Control appropriate room temperature and relative humidity. Light and darkness were alternated every 12 h. The experimental procedures received approval from the Institutional Animal Care and Use Committee of Nanjing University of Chinese Medicine (202306A074). At the end of the experiment, each rat was humanely euthanized via intraperitoneal injection with a 200 mg/kg dose of sodium pentobarbital solution.

### 2.2 Creation of the embolic stroke model

Embolic Stroke Modeling Methods were described by Zhang et al. (18). Rats were anesthetized with isoflurane (5% for induction and 2% for maintenance). A PE-50 tube is inserted into the donor rat’s femoral artery to procure arterial blood, which is subsequently maintained in a petri dish at 37°C for 2 h and at 4°C for 22 h. Transfer the clot to the PE-10 tube and aspirate and rinse the clot 10–15 times to eliminate red blood cells. The cleaned clot is gathered in a revised PE-50 catheter. The cervical vessels of rats were exposed after anesthesia. A modified PE-50 catheter that contained an embolus was inserted 19–22 mm from the external carotid artery to the internal carotid artery. Upon the catheter tip arriving at the source of the middle cerebral artery (MCA), it is retracted by 1–2 mm, and the thrombus is gradually injected into the MCA using 5–10 μL of saline. Extract the catheter 5 min post-injection. The laser speckle imaging system (RWD, China) was employed to assess brain blood flow, with successful occlusion indicated by a marked decrease in perfusion (Supplementary Figures 1A,B).

### 2.3 Experimental design and groups

The overall experiment consisted of the first and second experiments, with the rats randomized into multiple groups in each experiment.

1. In the initial trial, rats were randomly allocated into sham, model, 4.5 h rt-PA, 6 h rt-PA, acupuncture (A) + 4.5 h rt-PA, and A + 6 h rt-PA groups. At 4.5 h or 6 h following successful establishment of the embolic stroke model, rats received an injection of rt-PA via the tail vein (10 mg/kg), as per prior studies (19, 20).
2. To ascertain if acupuncture enhances the safety of delayed rt-PA thrombolysis through the mitophagy-NLRP3 inflammasome pathway, we conducted the second experimental trial. Rats were randomly allocated into sham, model, 6 h rt-PA, A + 6 h rt-PA, and A + 6 h rt-PA + mitophagy inhibitor (Mdivi-1) groups. At 6 h post-stroke, rats in the inhibitor group were injected intraperitoneally with Mdivi-1 (2 mg/kg) (21), while the remaining procedures for each group were consistent with those in the initial experiment.

### 2.4 Acupuncture treatment

The rats in the A + 6 h rt-PA and A + 6 h rt-PA + Mdivi-1 groups were treated with acupuncture at 6 h post-stroke. The rats in the A + 4.5 h rt-PA group were treated with acupuncture at 4.5 h post-stroke. Acupuncture points were selected as Shuigou (GV26, situated at the intersection of the superior and middle thirds of the human median sulcus) and bilateral Neiguan (PC6, positioned on the anterior forearm, 2 cm above the transverse line of the distal palmar wrist, between the palmar longus tendon and the radial carpal flexor tendon). Firstly, the bilateral PC6 acupoints were pricked straightly at a depth of 3 mm, and then the lift-insertion-twisting-laxation method was performed for a duration of 1 min; then the GV26 acupoint was pricked obliquely in the direction of the nasal septum for 2–3 mm, and then the sparrow-pecking needling was applied, and stimulation lasted for 1 min. Leave the needle in place for 30 min.

### 2.5 Evaluation of neurological performance

Neurological deficits were evaluated using a modified 6-point scale, with assessments conducted at 2 h and 24 h following stroke induction (22, 23). The criteria are the following: 0, no notable functional impairment; 1, flexion of the opposite forelimb; 2, diminished grip of the opposite forelimb during tail traction; 3, spontaneous, non-directional motion, exhibiting circling to the opposite side solely during tail traction; 4, spontaneous circling to the opposite side; 5, mortality. Neurologic deficit scores were required to reside between 2 and 4.

### 2.6 Assessment of infarction volume

24 h post-stroke, the rat brain tissues were immediately excised and subsequently fast frozen at −20°C refrigerator for 15 min. Frozen brain tissue was placed in a mold, and eight consecutive coronal brain slices, each approximately 2 mm thick, were cut 2 mm back from the frontal pole at equal intervals, followed by incubation with 2% TTC reagent (Sigma, USA) for approximately 15 min at 37°C in a dark environment. Normal regions are depicted in red, while ischemic regions are represented in white. The cerebral infarct volume percentage was ultimately computed using ImageJ software (version 1.5.4). The formula for calculating the volume of cerebral infarction is as follows: infarcted brain volume (%) = (total brain volume − stained brain volume) / total brain volume × 100%.

### 2.7 Assessment of BBB permeability

The rats were given 2% Evans Blue (EB, Sigma, USA) at a rat weight of 4 mL/kg via the tail vein for 2 h prior to euthanasia. The rats were perfused using saline to remove all EB from the organism. The brain was extracted from the cut skull, with the right brain soaked in formamide (1 mL/100 mg), and subsequently, the brain tissue was homogenized. Water bath at 60°C for 24 h, with subsequent centrifugation at 13000 rpm for 20 min. The absorbance was determined by measuring the optical density (OD) at a 620 nm wavelength with a spectrophotometer. The EB content was calculated using the formula: EB concentration (μg/mL) × volume of formamide (mL) / mass of brain tissue (g).

### 2.8 Evaluation of hemorrhagic transformation

HT following delayed rt-PA thrombolysis was assessed by quantifying hemoglobin levels in the ischemic brain hemisphere by spectrophotometry (24). Anesthetized rats underwent fast perfusion with saline, followed by excision of brain tissue from the ischemic hemisphere. Subsequent to weighing the tissue, 2 mL of pre-chilled 1 × PBS was included and homogenized. Utilize a centrifuge to separate and remove the supernatant. The hemoglobin concentration was tested using the QuantiChrom™ Hemoglobin Assay Kit (Hayward, USA). The OD value was measured using a microplate reader. The hemoglobin concentration (mg/dL) was calculated using the formula: (OD of Sample − OD of Blank) / (OD of Calibrator − OD of Blank) × 100.

### 2.9 Western blot analysis

Total protein was obtained from the tissue of the cerebral ischemia penumbra (IP). Protein concentration was calculated using the BCA Protein Assay Kit (Beyotime, China). The supernatant was combined with 5 × SDS-PAGE protein loading buffer and 1 × PBS to prepare the sample storage solution. Equal amounts were electrophoresed on an 8–12% SDS-PAGE gel and transferred to PVDF membranes (Millipore, USA). The following particular primary antibodies were added to the membrane after it had been blocked with 5% BSA for 2 h at room temperature and then incubated for the entire night at 4°C: Pink1 (1:1000, ab186303, Abcam, UK), Parkin (1:1000, ab77924, Abcam, UK), LC3B (1:2000, ab192890, Abcam, UK), P62 (1:2000, ab109012, Abcam, UK), COX4I1 (1:1000, ab14744, Abcam, UK), NLRP3 (1:1000, ab263899, Abcam, UK), Caspase-1 (1:1000, ab286125, Abcam, UK), and ZO-1 (1:2000, 21,773-1-AP, Proteintech, China). Following three washes, the membrane was incubated for 1 h at room temperature with HRP-conjugated goat anti-rabbit (1:10000, RS0001, Immunoway, China) or anti-mouse IgG (1:10000, RS0002, Immunoway, China). The bands were ultimately identified using improved chemiluminescence. *β*-tubulin (1:10000, 30302ES20, Yeasen, China) and GAPDH (1:10000, 10,419-AP, Proteintech, China) were used as internal controls. ImageJ software was used to quantify the grayscale values of the target bands and the internal reference bands. The ratios of the internal reference bands to the destination bands were normalized with a sham group mean of 1 to improve the accuracy and comparability of the data.

### 2.10 Real-time PCR

Total mRNA was extracted from brain tissue with Trizol and subsequently reverse transcribed into cDNA following the kit instructions (Yeasen, China). SYBR Green Master Mix was used for real-time PCR. A three-step PCR amplification program was selected: 95°C for 5 min of pre-denaturation, 95°C for 10 s of denaturation, 55°C for 10 s of annealing, and 72°C for 20 s of extension, comprising a total of 40 cycles. The 2^-ΔΔCt^ experiment was performed to statistically ascertain the levels of mRNA expression. Additionally, *β*-tubulin was utilized as an internal reference. The primers utilized are displayed in Table 1.

**Table 1:** Primers for real-time PCR.

Gene	Primer	Sequences (5′ to 3′)
Pink1	F	GAAGCCACCATGCCCACACTG
	R	CATCTGCTCCCTTTGAGACGACATC
Parkin	F	CCAACCTCAGACAAGGACACATCAG
	R	TGGCGGTGGTTACATTGGAAGAC
LC3	F	CAAGCCTTCTTCCTCCTGGTGAATG
	R	AGTGCTGTCCCGAACGTCTCC
P62	F	GAAAGAGCGGGTACTGATCCC
	R	CCATAGCATGGGCCATAAGAG
COX4I1	F	TGAGATGAACAAGGGCACCAATGAG
	R	GCCACCCAGTCACGATCAAAGG
NLRP3	F	CTGCTGTGCGTGGGACTGAAG
	R	AGAACCAATGCGAGATCCTGACAAC
Caspase-1	F	AAACACCCACTCGTACACGTCTTG
	R	AGGTCAACATCAGCTCCGACTCTC
ZO-1	F	GCCAAGCCAGTCCATTCTCAGAG
	R	TCCATAGCATCAGTTTCGGGTTTCC
β-tubulin	F	CAATGAGGCCTCCTCTCACA
	R	TGTATAGTGCCCTTTGGCCC

### 2.11 Enzyme-linked immunosorbent assay

IL-18 and IL-1β levels were measured by ELISA. Rat cerebral cortex tissue was homogenized on ice and centrifuged at 3000 rpm for 20 min at 4°C, followed by extraction of the supernatants. An enzyme calibrator (Elabscience, China) was used to assess the OD of each well at 450 nm. The level of the target molecule was measured based on the standard curve derived from the diluted standards.

### 2.12 Transmission electron microscopy

The cortex IP area of rats, perfused through the heart, was sectioned into 3 small cubes measuring 1 mm × 1 mm × 1 mm and promptly preserved with 2.5% glutaraldehyde for 24 h at 4°C. The slice thickness was determined to be 60 nm. Subsequently, double staining was conducted utilizing 3% uranyl acetate and lead citrate. TEM was used to observe the architecture of the BBB as well as neuronal mitochondria.

### 2.13 Statistical analysis

The data were indicated as mean ± standard deviation (mean ± SD). The analysis was performed using GraphPad Prism version 8.0.2. When the data for each group followed a normal distribution, the one-way ANOVA was employed for multiple group analyses. When the data did not meet the criteria for distribution as normal, a nonparametric test was utilized. Significant differences were deemed to be *p* < 0.05.

## 3 Results

### 3.1 Acupuncture reduced HT and improved brain injury caused by delayed rt-PA thrombolysis after ischemic stroke

**Figure 1:**
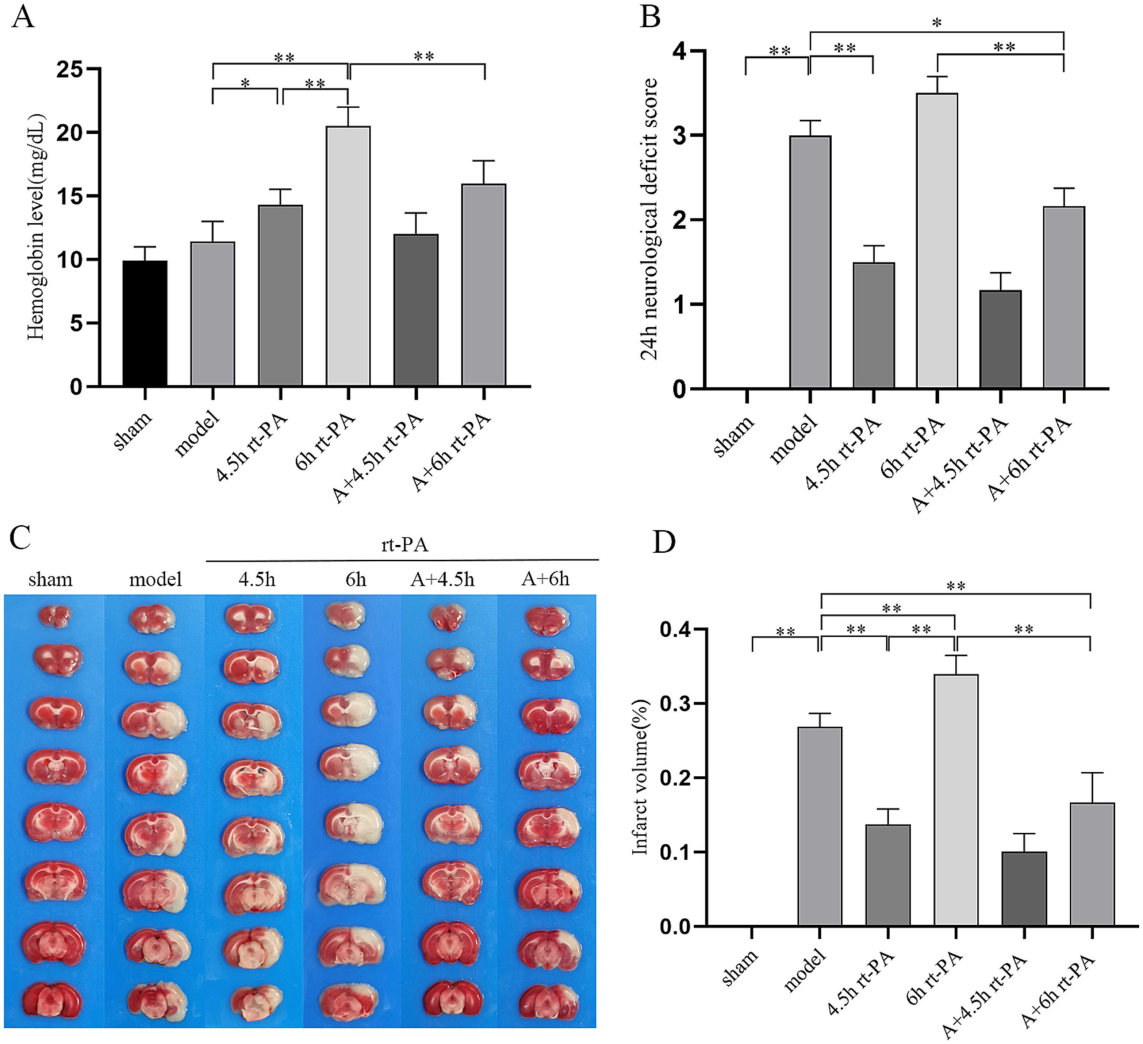
Acupuncture reduced HT and improved brain damage. **(A)** HT in each group (*n* = 6). **(B)** 24 h post-stroke Bederson test (*n* = 12). **(C)** Photographs of representative brain sections with TTC staining. **(D)** Volume of infarcted brain (*n* = 6). *p < 0.05; **p < 0.01.

We evaluated the efficacy of acupuncture on delayed rt-PA-induced complications by evaluating HT 24 h post-stroke. The hemoglobin levels in both the 4.5 h and 6 h rt-PA groups exceeded those of the model group, with a particularly notable increase in the 6 h rt-PA group beyond the time window. However, in comparison with the 6 h rt-PA group, the hemoglobin content was markedly reduced in the A + 6 h rt-PA group (Figure 1A).

In addition, we assessed the impact of acupuncture on delayed rt-PA treatment for acute cerebral ischemia by measuring the neurological impairment score at 2 and 24 h post-stroke, as well as evaluating the volume of cerebral infarct volume at 24 h after stroke. The neurologic impairment score was reduced in the A + 6 h rt-PA group relative to the model and 6 h rt-PA groups at 24 h after stroke (Figure 1B). Compared to the model group, the volume of cerebral infarction was decreased in the 4.5 h rt-PA group and increased in the 6 h rt-PA group. However, the volume of cerebral infarction in the A + 6 h rt-PA group was considerably reduced relative to the 6 h rt-PA group (Figures 1C,D).

### 3.2 Acupuncture diminished BBB permeability and elevated expression of TJ proteins following delayed rt-PA thrombolysis after ischemic stroke

**Figure 2:**
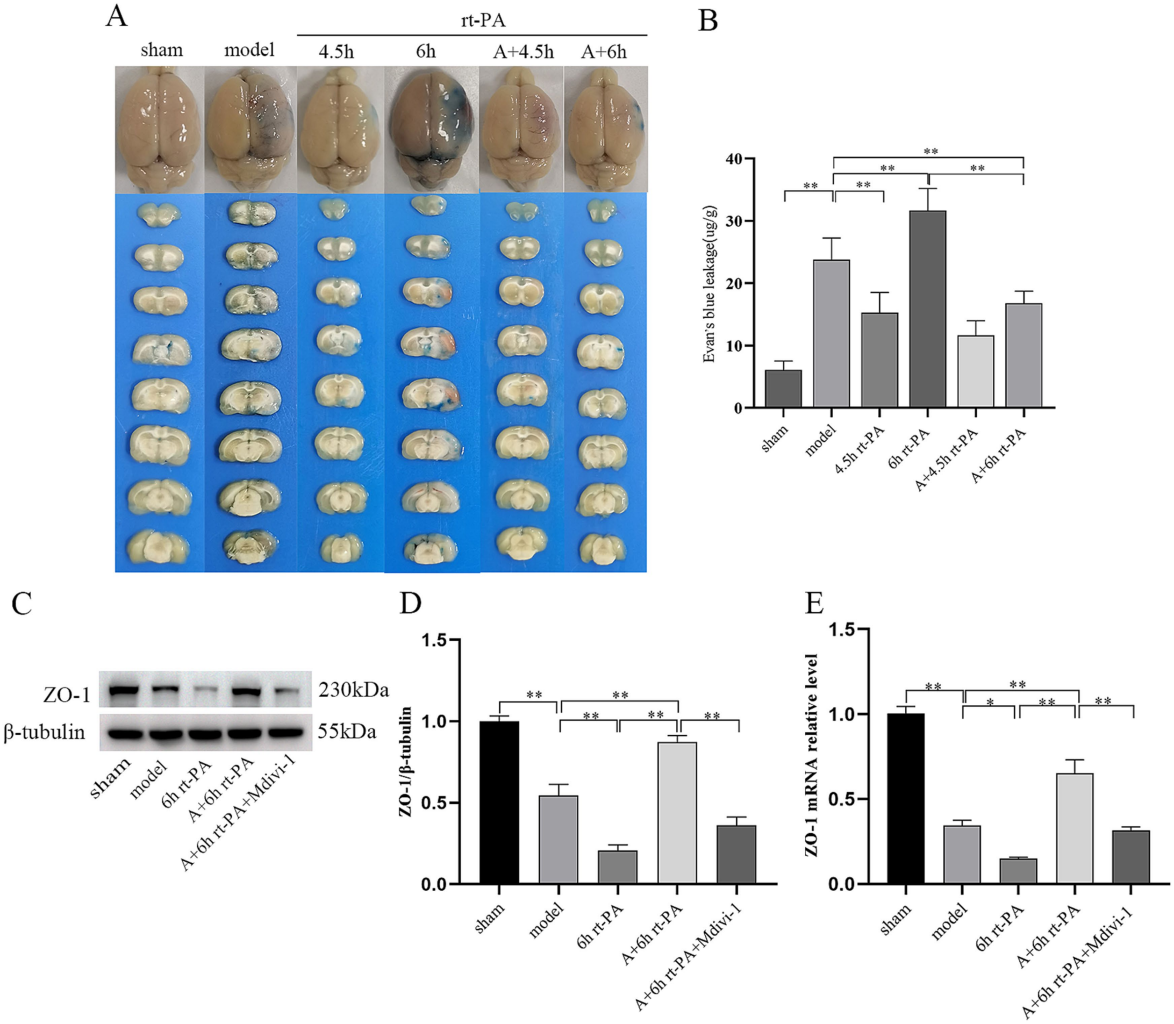
Acupuncture diminished BBB permeability and elevated expression of TJ proteins. **(A)** Graphical representation of EB penetration in each group of rats. **(B)** Permeability of the BBB in each group (*n* = 6). **(C)** ZO-1 protein western blot graph. **(D)** Relative concentrations of the ZO-1 protein (*n* = 3). **(E)** Relative concentrations of ZO-1 mRNA (*n* = 6). *p < 0.05; **p < 0.01.

To evaluate the permeability of the BBB, we measured the amount of EB leakage. EB leakage was significantly increased in the model group in comparison to the sham group and further elevated in the 6 h rt-PA group. However, in contrast to the model group and the 6 h rt-PA group, EB levels were markedly diminished in the A + 6 h rt-PA group (Figures 2A,B).

Furthermore, considering the significant function of tight junctions (TJs) in preserving the integrity of the BBB, we studied whether acupuncture elevated the expression of ZO-1 in rt-PA-treated thromboembolic stroke rats. Compared with the sham group, the protein and mRNA levels of ZO-1 were dramatically reduced in the model group. Furthermore, these levels were further decreased in the 6 h rt-PA group compared to the model group. However, in the A + 6 h rt-PA group, the protein and mRNA expression of ZO-1 was distinctly enhanced in contrast to the model and the 6 h rt-PA groups. In addition, the inhibitor group showed the opposite trend to acupuncture (Figures 2C–E).

### 3.3 Acupuncture ameliorated the disruption of BBB caused by delayed rt-PA thrombolysis after ischemic stroke

**Figure 3:**
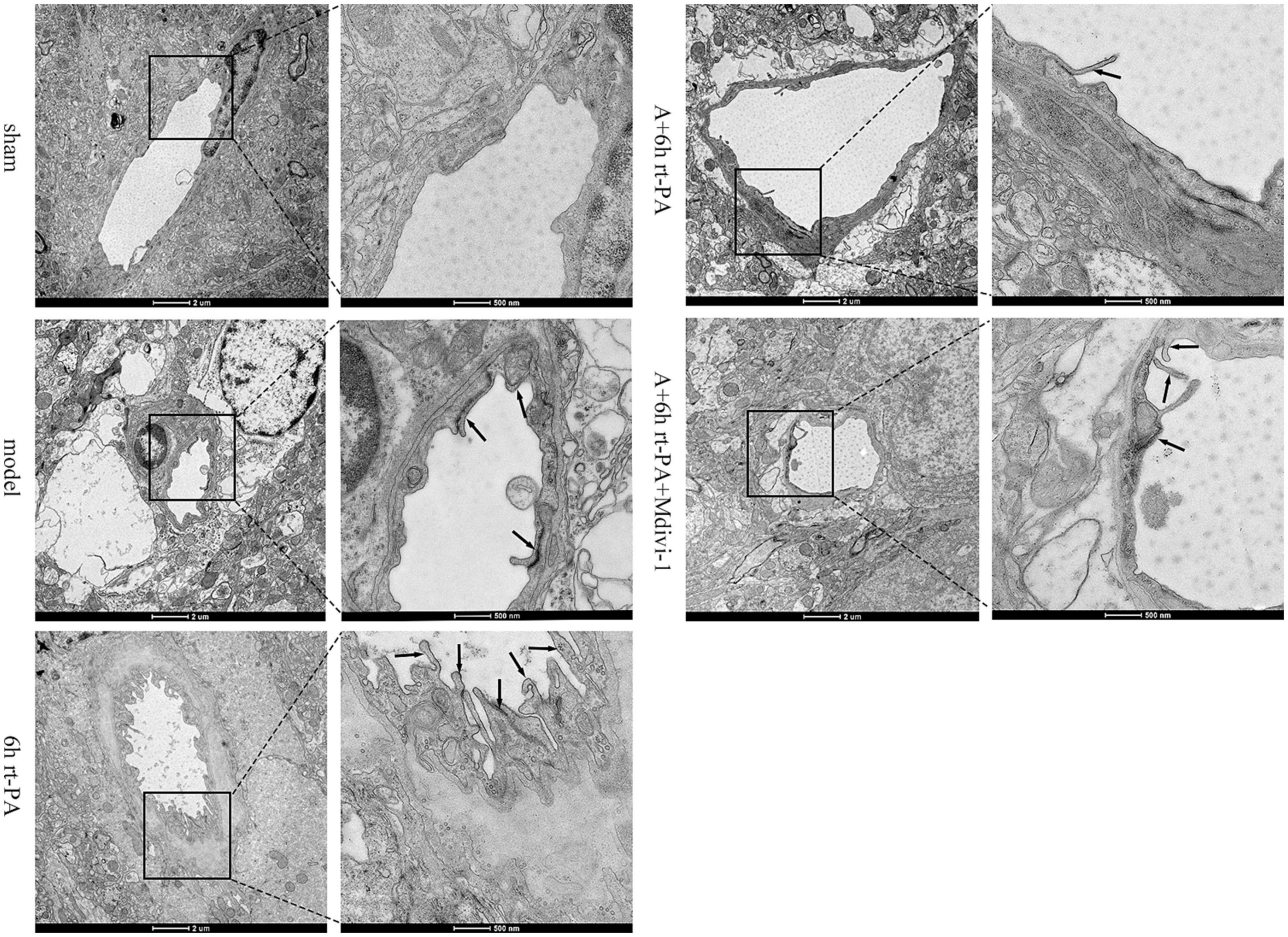
BBB ultrastructural TEM results. Scale bar at low magnification = 2 μm, scale bar at high magnification = 500 nm. Black arrows denote damaged tight junctions.

BBB ultrastructural morphology of the sham group was normal, and the surface of endothelial cells was smooth and intact, continuous and regular, and the tight junction structure was dense and sturdy. In comparison with the sham group, endothelial cells in the model group were swollen, deformed and irregular, with disrupted tight junctions and loose connections. The swelling and deformation of endothelial cells in the BBB of the 6 h rt-PA group were further aggravated compared with that of the model group, and the tight junctions were broken, and the ultrastructure of the BBB was severely damaged. As compared with the 6 h rt-PA group, the ultrastructural disruption of BBB in the A + 6 h rt-PA group was attenuated, and its endothelial cell morphology was regular and basically continuous and intact, and the disruption of tight junctions was significantly reduced. However, the inhibitor effectively antagonized the effect of acupuncture (Figure 3).

### 3.4 Acupuncture inhibited overexpression of NLRP3 inflammasome-related biomarkers caused by delayed rt-PA thrombolysis after ischemic stroke

**Figure 4:**
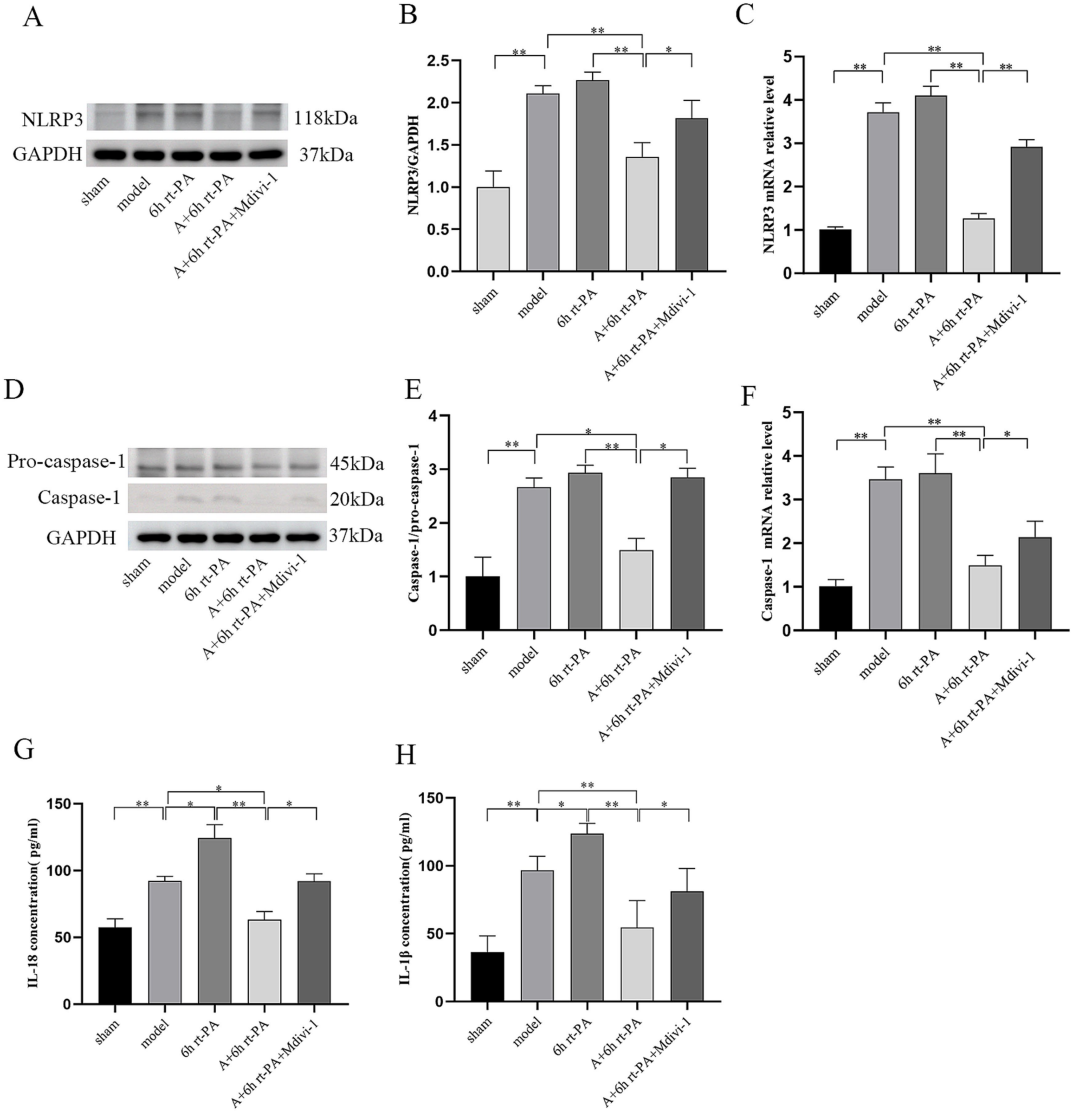
Comparison of the expression of NLRP3 inflammasome-related biomarkers in rats of various groups. **(A,D)** NLRP3 and Caspase-1 protein western blot graphs (*n* = 3); **(B)** NLRP3 protein expression quantitative statistics; **(C)** NLRP3 mRNA expression quantitative statistics (*n* = 6); **(E)** Caspase-1 protein expression quantitative statistics. **(F)** Caspase-1 mRNA expression quantitative statistics (*n* = 6); **(G)** content quantification of IL-18 (*n* = 6); **(H)** content quantification of IL-1*β* (*n* = 6). *p < 0.05; **p < 0.01.

Inflammasome activation is a major contributor to BBB disruption, and the release of mediators can incite a profound inflammatory response. The results indicated a substantial increase in the expressions of NLRP3 and mature Caspase-1 in the model and the 6 h rt-PA group in contrast to the sham group; however, the expression of NLRP3 and mature Caspase-1 was significantly lower in the A + 6 h rt-PA group compared to the model and 6 h rt-PA groups. Meanwhile, the expression of NLRP3 and mature Caspase-1 was again enhanced in the A + 6 h rt-PA + Mdivi-1 group than in the A + 6 h rt-PA group (Figures 4A–F). Furthermore, the results for IL-18 and IL-1β showed approximately the same trend (Figures 4G,H).

### 3.5 Acupuncture enhanced the level of mitophagy following delayed rt-PA thrombolysis after ischemic stroke

**Figure 5:**
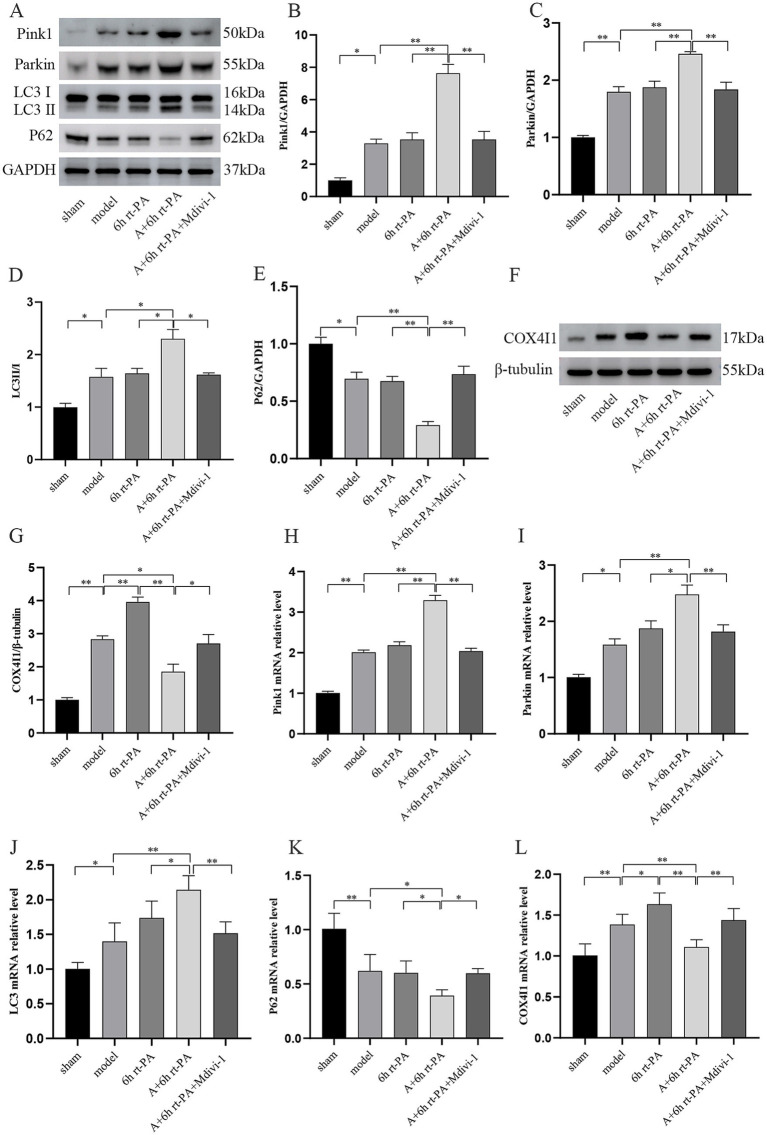
Comparison of mitophagy-related protein and mRNA expression in rats of various groups. **(A,F)** Western blot bands of mitophagy-related proteins in each group of rats; **(B–E,G)** Pink1, Parkin, LC3, P62, COX4I1 protein expression quantitative statistics (*n* = 3); **(H–L)** Pink1, Parkin, LC3, P62, COX4I1 mRNA expression quantitative statistics (*n* = 6). *p < 0.05; **p < 0.01.

The levels of mitophagy were evaluated using western blot and real-time PCR. Compared to the sham group, the levels of Pink1, Parkin, and LC3 protein and mRNA were significantly elevated in both the model and 6 h rt-PA groups. Furthermore, these levels were further increased in the A + 6 h rt-PA group compared to the model and 6 h rt-PA groups. However, there was a decreasing trend in the levels of Pink1, Parkin, LC3 protein and mRNA in the A + 6 h rt-PA + Mdivi-1 group relative to the A + 6 h rt-PA group (Figures 5A–D, H–J). Furthermore, P62 expression was markedly diminished in both the model and 6 h rt-PA groups compared to the sham group. P62 expression was further diminished in the A + 6 h rt-PA group. However, the P62 expression in the A + 6 h rt-PA + Mdivi-1 group was opposite to that in the A + 6 h rt-PA group (Figures 5E,K).

As a mitochondrial intima protein, COX4I1 can effectively reflect mitochondrial damage after acute cerebral infarction. The expression of COX4I1 was increased in the model and 6 h rt-PA groups compared to the sham group and was higher in the 6 h rt-PA group with a significant difference in the model group. While the expression of COX4I1 decreased in the A + 6 h rt-PA group compared with the model and 6 h rt-PA groups. It was also observed that the COX4I1 expression level in the A + 6 h rt-PA + Mdivi-1 group was again enhanced compared with that in the A + 6 h rt-PA group (Figures 5F–G,L).

### 3.6 Acupuncture alleviated mitochondrial morphology disruption of neurons following delayed rt-PA thrombolysis after ischemic stroke

**Figure 6:**
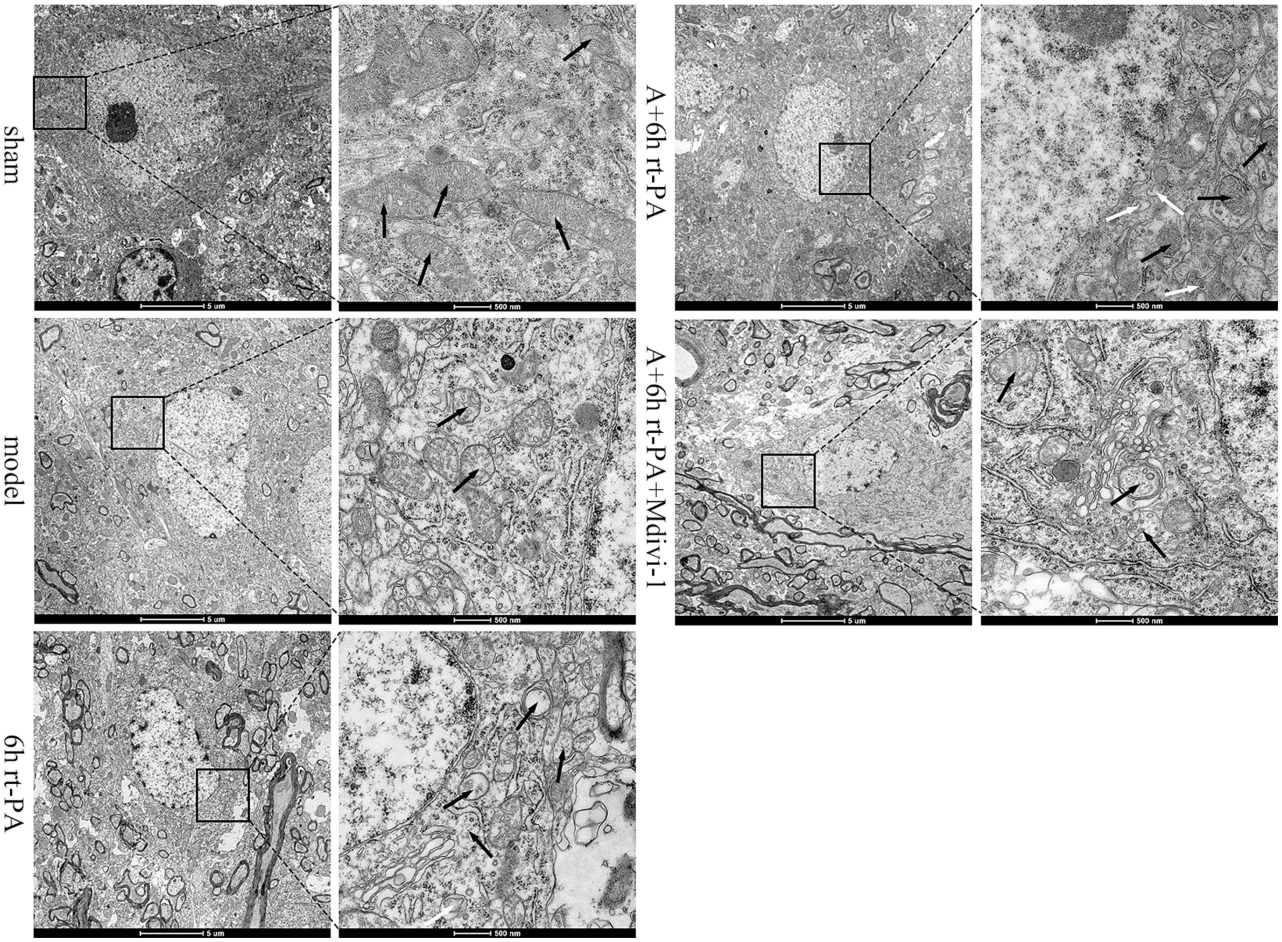
Ultrastructural TEM results of neuronal mitochondria. Scale bar at low magnification = 5 μm, scale bar at high magnification = 500 nm. Black arrows denote mitochondria; white arrows signify autophagosomes of mitochondria.

We used TEM to examine the morphological alterations of the mitochondria in the neurons of each group. The mitochondrial structure of the sham group was normal, with a clear double-layered membrane and mitochondrial cristae. Compared with the sham group, the mitochondrial structure of the model group was damaged, the double-layered membrane structure was unclear or even disappeared, and the mitochondrial cristae were broken. In comparison with the model group, the mitochondrial cristae of the 6 h rt-PA group were broken or even completely disappeared, the mitochondria were swollen into vacuoles and a small number of mitochondrial autophagic vesicles could be seen. However, relative to the 6 h rt-PA group, the mitochondria in the A + 6 h rt-PA group were less disrupted, the bilayer membrane structure and cristae were clear and intact, and a greater number of mitochondrial autophagosomes were seen. The mitochondrial structure of the A + 6 h rt-PA + Mdivi-1 group was still disrupted, the cristae integrity was missing and the bilayer membrane structure was blurred when compared with the A + 6 h rt-PA group (Figure 6).

## 4 Discussion

**Figure 7:**
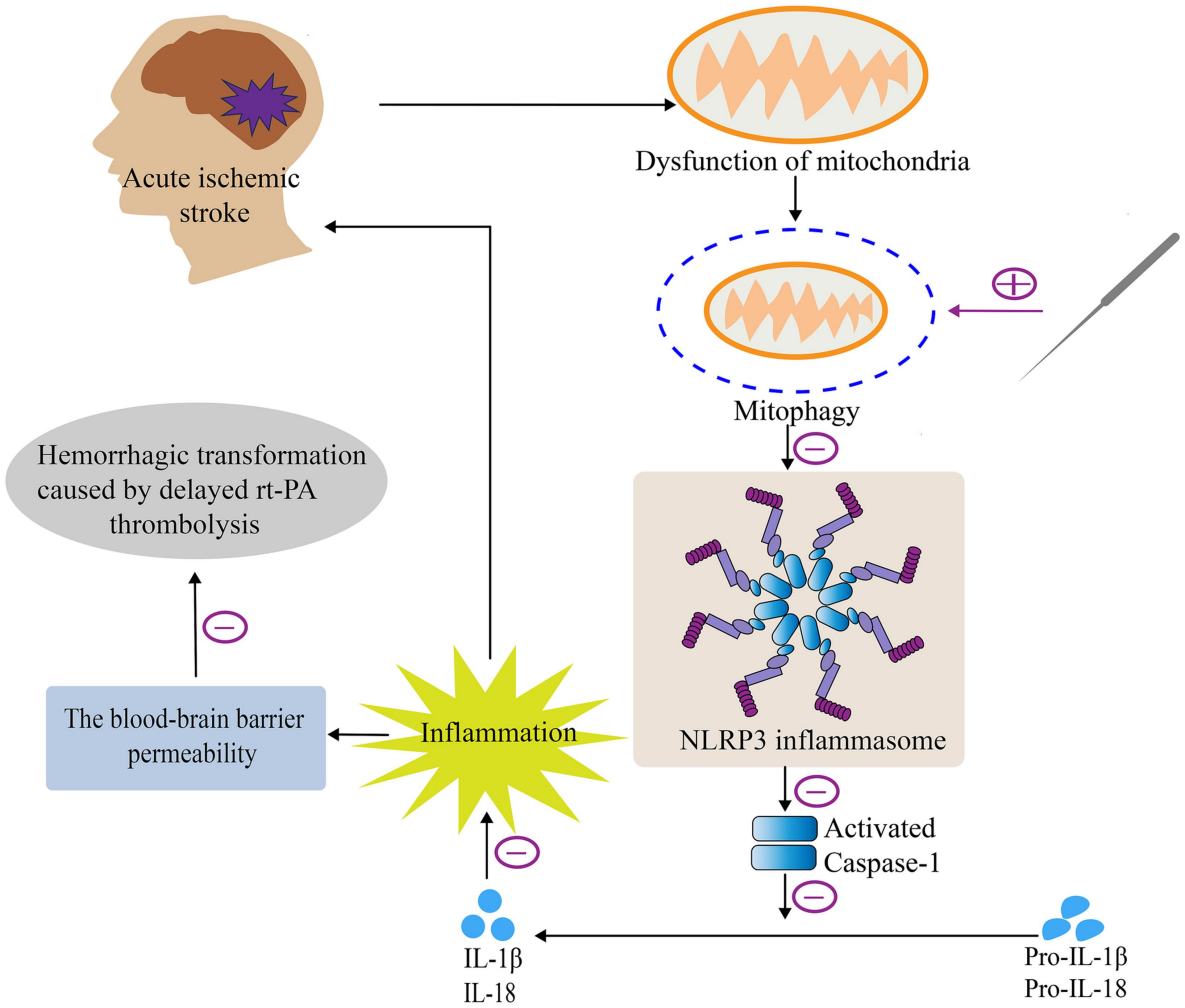
Mechanism of acupuncture in attenuating HT after delayed rt-PA treatment in acute cerebral infarction. Briefly, acupuncture alleviates HT after delayed rt-PA treatment in acute cerebral infarction via regulation of the mitophagy-NLRP3 inflammasome pathway.

In this study, first we verified that acupuncture can enhance neurological deficit scores and decrease the brain infarct volume and BBB permeability after delayed rt-PA thrombolysis in rats with acute ischemic stroke, which in turn attenuates the risk of post-thrombolytic HT and exerts a cerebral protective effect. Subsequently, we focused on exploring the intrinsic mechanisms of acupuncture exerting cerebral protective effects, that is, whether acupuncture has an effect on mitophagy or NLRP3 inflammasome and what correlation exists between the both of them. The results suggested that acupuncture could alleviate HT after delayed rt-PA therapy for acute cerebral ischemia through a pathway that promotes mitophagy and suppresses the activity of the NLRP3 inflammasome, improving the safety of delayed rt-PA thrombolysis (Figure 7).

Acupuncture is an important component of traditional Chinese medicine and has long been used in the treatment of stroke (25). Xingnao Kaiqiao (XNKQ) acupuncture was founded by Prof. Xuemin Shi, and is highly effective in the treatment of stroke, especially ischemic stroke (26). GV26 and PC6 are the main acupoints for XNKQ acupuncture. GV26 is located in the governor vessel, one of the key points for first aid, which can diastole cerebral blood vessels, improve collateral circulation, increase cerebral blood supply, and can effectively improve neurological function. PC6 is associated with the heart by traveling up the hand syncopal pericardium meridian, which can improve the cardiac output of stroke patients and increase the blood supply to the brain (27). It has been shown that XNKQ acupuncture is safe and effective in the treatment of acute ischemic stroke (28). Consequently, the GV26 and PC6 acupoints were chosen for acupuncture for acute ischemic stroke.

Mitochondria possess a distinctive double membrane structure and exhibit differential permeability between the interior and exterior membranes, resulting in a trans-mitochondrial membrane potential essential for appropriate mitochondrial function (29). If the mitochondria are exposed to external stimuli, such as the occurrence of acute stroke, a series of unbalanced reactions occurs, and if peroxidation is increased *in vivo* and exceeds the body’s antioxidant capacity, oxidative stress will arise (30). Under ischemic and hypoxic conditions, brain tissue experiences a reduction in mitochondrial metabolism and ATP synthesis. Meanwhile, the electron transport chain of mitochondria is also destroyed. During the reperfusion phase, impaired mitochondria can generate excess reactive oxygen species (ROS) from a variety of sources, mainly from ETCs (31). If the excess mitochondrial ROS cannot be removed in time, it will cause changes in mitochondrial membrane permeability and structural damage, which ultimately leads to mitochondrial dysfunction (32). Thus, timely removal of dysfunctional mitochondria from the cell is of critical importance to ensure mitochondrial quality and maintain cellular homeostasis. Mitophagy could selectively remove incomplete or damaged mitochondria to preserve the functional integrity of that network of mitochondria (33). It has been shown that the ubiquitin-mediated pathway is an important pathway for mitophagy-inducing signaling and that the Pink1/Parkin pathway is the predominant form (31, 34). Pink1 is a mitochondrial serine/threonine kinase encoded by a nucleic acid that can mediate substrate ubiquitination (35). Pink1 is produced in the cytoplasm and transported to the interior of the mitochondria via outer and inner mitochondrial membrane transporter enzymes before being degraded by matrix proteolytic enzymes (36). Once mitochondria are destroyed, the structure of the bilayer membrane is altered, and depolarization occurs due to changes in membrane permeability and membrane potential, Pink1 is incapable of entering the mitochondrial matrix and accomplishing protein degradation normally from the exterior membrane via the membrane gap. As a result, Pink1 accumulates in large amounts and is activated by phosphorylation. Parkin is an E3 ubiquitin ligase encoded by the PARK2 gene (37, 38). At this point, phosphorylation-activated Pink1 couples Parkin to the mitochondrial external membrane and promotes its activity (39, 40). Activated Parkin ubiquitinates several proteins of the mitochondrial outer membrane and forms ubiquitin chains to activate the ubiquitin protease system, thereby initiating mitophagy by recruiting the autophagy marker LC3 to the mitochondria (41). Furthermore, the autophagy junction protein P62/SQSTM1 recognizes Parkin and the ubiquitin chains formed by its ubiquitinated proteins and binds specifically to LC3 on the membrane surface of the autophagosome. The autophagosome membrane envelops mitochondria to form mitochondrial autophagosomes and fuses with lysosomes to form mitochondrial autophagy lysosomes, which ultimately remove damaged mitochondria (9). This experiment showed that the levels of mitophagy proteins and mRNA expression levels, such as Pink1, were markedly elevated, while the expression level of P62 showed a decreasing tendency after delayed rt-PA thrombolysis combined with acupuncture intervention in rats with a cerebral infarction model. Additionally, the use of mitophagy inhibitors tended to reverse these observed trends. Furthermore, the ultrastructure of neuronal mitochondria showed that after acupuncture intervention was given on the basis of delayed rt-PA thrombolysis, mitochondrial disruption was attenuated, with clear and intact bilayer membrane structure and cristae, without swelling, deformation, and vacuoles, and more mitochondrial autophagosomes appeared. These results collectively suggest that acupuncture could increase mitochondrial autophagic activity in neurons of a thromboembolic rat model. It has also been noted that administration of a mitophagy inhibitor intervention reversed the protective effects of treatment, aligning with the findings of the current study (42).

The inflammasome is pivotal in the process of brain ischemia damage, which has attracted considerable interest (43–45). Recent years have witnessed an increase in investigations into the NLRP3 inflammasome, the importance of which cannot be overstated (45, 46). The evidence suggests that mitochondria have a tight relationship with the inflammasome (47). The mitochondrial injury generates a large amount of ROS into the cytosol, which induces the activation of the NLRP3 inflammasome (48). The NLRP3 inflammasome further exacerbates the inflammatory response by forming active IL-18 and IL-1β through a series of processes and releasing them extracellularly (49). Some studies implicate that the activated NLRP3 inflammasome and its subsequent inflammatory mediators have a role in cerebral ischemia damage (50–52). Pink1/Parkin mitophagy is a critical pathway for the inhibition of the NLRP3 inflammasome (53). Current work has examined the impact of acupuncture-induced mitophagy on the NLRP3 inflammasome and its associated inflammatory mediators during ischemic brain injury. The study results showed acupuncture could decrease the level of NLRP3 and its downstream factors, which indicated that acupuncture could effectively suppress the NLRP3 inflammasome activity. In parallel, it was confirmed that melatonin upregulated mitophagy, and NLRP3 inflammasome activation was effectively suppressed (54). This aligns with the findings of the current investigation.

The European Cooperative Acute Stroke Study (ECASS)-II categorized HT into hemorrhagic infarcts (HI) and parenchymal hematomas (PH) (55). Recent epidemiological data showed that the incidence of HT was 17.8% in patients treated with intravenous thrombolysis (56). Substantial evidence demonstrated a robust association between the risk of HT and several factors, predominantly including delayed thrombolysis-reperfusion therapy, higher National Institutes of Health Stroke Scale (NIHSS), atrial fibrillation, hypertension, moderate-to-severe cerebral leukoaraiosis, and large cerebral infarcts (57, 58). Furthermore, patients with elevated blood glucose levels are also related to an exacerbated risk of HT (59). Among these factors, delayed rt-PA thrombolysis (after 4.5 h of stroke onset) is a significant cause of an increased risk of HT (60). Studies have identified that impairment of the BBB integrity plays an essential role in HT pathological changes (61). In fact, the integrity and permeability of the BBB are slightly disturbed shortly after the beginning of an ischemic stroke (62). And delayed rt-PA treatment following a stroke exacerbates BBB damage and elevates the likelihood of HT (63). Considerable studies support that the NLRP3 inflammasome is active in ischemic stroke (64, 65), inducing an inflammatory response that may be linked to BBB disruption (66). Inflammatory infiltration leads to structural disruption and dysfunction of the BBB, TJ proteins are no longer dense, and gaps are enlarged, which expands the BBB’s permeability (67). This causes the entry of peripheral inflammatory substances into the cerebral tissue, further amplifying the inflammatory response. What’s more, when delayed rt-PA thrombolysis is used, it will aggravate the inflammatory response, exacerbate BBB destruction, and heighten the likelihood of HT. Consequently, inhibiting NLRP3 inflammasome activity has become a viable therapeutic approach for thrombolysis in acute cerebral infarction. In the current work, following 6 h of thrombolysis combined with acupuncture intervention, the level of ZO-1 protein and mRNA considerably increased; however, the mitochondrial inhibitor reversed the effect of acupuncture. We also observed the ultrastructural changes of the BBB using TEM, and acupuncture could attenuate the ultrastructural destruction of the BBB as well as improve the continuity and integrity of its morphology.

Currently, only 5.64% of ischemic stroke patients in China receive rt-PA thrombolysis due to the limited 4.5 h time window for rt-PA thrombolysis in cerebral infarction and complications such as severe HT (68). Past investigations have evidenced that selecting patients to start treatment between 4.5–6 h based on simple indicators such as the modified Rankin score (mRS) fails to provide a population in which the effects of rt-PA are safe and effective (69). However, our previous study showed that acupuncture could extend the time window for thrombolysis in embolic model rats to 6 h (27). This current study further validated that acupuncture could effectively reduce HT following intravenous rt-PA thrombolysis in embolic model rats beyond the time window, thereby enhancing the safety of thrombolysis. A prospective cohort study suggested that acupuncture not only reduces the incidence of HT in patients with rt-PA intravenous thrombolysis for cerebral infarction within the time window, but also exerts a positive effect on decreasing the disability rate of the patients and improving the ability of daily life (70). More patients will benefit if the results of the basic study of acupuncture to reduce HT in thrombolysis outside the time window are applied to clinical practice. Future research should focus on elucidating the detailed mechanisms and optimizing the timing and application of acupuncture in the treatment of ischemic stroke.

## 5 Conclusion

In conclusion, the study concluded that acupuncture could enhance Pink1/Parkin-mediated mitophagy and suppress the NLRP3 inflammasome activity, thereby reducing HT after delayed rt-PA treatment in acute embolic stroke. We expect that this finding could contribute to reliable theoretical support for clinical thrombolysis for acute cerebral ischemia.

## Data Availability

The raw data supporting the conclusions of this article will be made available by the authors, without undue reservation.

## References

[1] Tu WJ Zhao Z Yin P Cao L Zeng J Chen H. Estimated burden of stroke in China in 2020. JAMA Netw Open. (2023) 6:e231455. doi: 10.1001/jamanetworkopen.2023.1455, PMID: 36862407 PMC9982699

[2] Powers WJ Rabinstein AA Ackerson T Adeoye OM Bambakidis NC Becker K. Guidelines for the early Management of Patients with Acute Ischemic Stroke: 2019 update to the 2018 guidelines for the early Management of Acute Ischemic Stroke: a guideline for healthcare professionals from the American Heart Association/American Stroke Association. Stroke. (2019) 50:e344–418. doi: 10.1161/STR.0000000000000211, PMID: 31662037

[3] Bar B Biller J. Select hyperacute complications of ischemic stroke: cerebral edema, hemorrhagic transformation, and orolingual angioedema secondary to intravenous Alteplase. Expert Rev Neurother. (2018) 18:749–59. doi: 10.1080/14737175.2018.1521723, PMID: 30215283

[4] Lindley RI Wardlaw JM Sandercock PA Rimdusid P Lewis SC Signorini DF. Frequency and risk factors for spontaneous hemorrhagic transformation of cerebral infarction. J Stroke Cerebrovasc Dis. (2004) 13:235–46. doi: 10.1016/j.jstrokecerebrovasdis.2004.03.003 17903981

[5] Han PP Han Y Shen XY Gao ZK Bi X. NLRP3 inflammasome activation after ischemic stroke. Behav Brain Res. (2023) 452:114578. doi: 10.1016/j.bbr.2023.114578 37437697

[6] Ozaki E Campbell M Doyle SL. Targeting the NLRP3 inflammasome in chronic inflammatory diseases: current perspectives. J Inflamm Res. (2015) 8:15–27. doi: 10.2147/JIR.S51250, PMID: 25653548 PMC4303395

[7] Shao A Gao S Wu H Xu W Pan Y Fang Y. Melatonin ameliorates hemorrhagic transformation via suppression of ROS-induced NLRP3 activation after cerebral ischemia in hyperglycemic rats. Oxidative Med Cell Longev. (2021) 2021:6659282. doi: 10.1155/2021/6659282, PMID: 33777317 PMC7972845

[8] Wen H Miao EA Ting JP. Mechanisms of NOD-like receptor-associated inflammasome activation. Immunity. (2013) 39:432–41. doi: 10.1016/j.immuni.2013.08.037, PMID: 24054327 PMC3835203

[9] Lazarou M. Keeping the immune system in check: a role for mitophagy. Immunol Cell Biol. (2015) 93:3–10. doi: 10.1038/icb.2014.75, PMID: 25267485

[10] Shen Z Zheng Y Wu J Chen Y Wu X Zhou Y. PARK2-dependent mitophagy induced by acidic postconditioning protects against focal cerebral ischemia and extends the reperfusion window. Autophagy. (2017) 13:473–85. doi: 10.1080/15548627.2016.1274596, PMID: 28103118 PMC5361599

[11] Zhang X Yuan Y Jiang L Zhang J Gao J Shen Z. Endoplasmic reticulum stress induced by tunicamycin and thapsigargin protects against transient ischemic brain injury: involvement of PARK2-dependent mitophagy. Autophagy. (2014) 10:1801–13. doi: 10.4161/auto.32136, PMID: 25126734 PMC4198364

[12] Koyano F Okatsu K Kosako H Tamura Y Go E Kimura M. Ubiquitin is phosphorylated by PINK1 to activate parkin. Nature. (2014) 510:162–6. doi: 10.1038/nature13392 24784582

[13] Ma S Chen J Feng J Zhang R Fan M Han D. Melatonin ameliorates the progression of atherosclerosis via Mitophagy activation and NLRP3 Inflammasome inhibition. Oxidative Med Cell Longev. (2018) 2018:9286458. doi: 10.1155/2018/9286458, PMID: 30254716 PMC6142770

[14] Mai CT Wu MM Wang CL Su ZR Cheng YY Zhang XJ. Palmatine attenuated dextran sulfate sodium (DSS)-induced colitis via promoting mitophagy-mediated NLRP3 inflammasome inactivation. Mol Immunol. (2019) 105:76–85. doi: 10.1016/j.molimm.2018.10.015, PMID: 30496979

[15] He Q Li Z Meng C Wu J Zhao Y Zhao J. Parkin-dependent Mitophagy is required for the inhibition of ATF4 on NLRP3 Inflammasome activation in cerebral ischemia-reperfusion injury in rats. Cells. (2019) 8:897. doi: 10.3390/cells8080897, PMID: 31416289 PMC6721752

[16] Sha R Zhang B Han X Peng J Zheng C Zhang F. Electroacupuncture alleviates ischemic brain injury by inhibiting the miR-223/NLRP3 pathway. Med Sci Monit. (2019) 25:4723–33. doi: 10.12659/MSM.917213, PMID: 31237865 PMC6607941

[17] Wang H Chen S Zhang Y Xu H Sun H. Electroacupuncture ameliorates neuronal injury by Pink1/Parkin-mediated mitophagy clearance in cerebral ischemia-reperfusion. Nitric Oxide. (2019) 91:23–34. doi: 10.1016/j.niox.2019.07.004, PMID: 31323277

[18] Zhang L Zhang RL Jiang Q Ding G Chopp M Zhang ZG. Focal embolic cerebral ischemia in the rat. Nat Protoc. (2015) 10:539–47. doi: 10.1038/nprot.2015.036, PMID: 25741989 PMC4602402

[19] Si Z Liu J Hu K Lin Y Liu J Wang A. Effects of thrombolysis within 6 hours on acute cerebral infarction in an improved rat embolic middle cerebral artery occlusion model for ischaemic stroke. J Cell Mol Med. (2019) 23:2468–74. doi: 10.1111/jcmm.14120, PMID: 30697923 PMC6433693

[20] Yamashita T Kamiya T Deguchi K Inaba T Zhang H Shang J. Dissociation and protection of the neurovascular unit after thrombolysis and reperfusion in ischemic rat brain. J Cereb Blood Flow Metab. (2009) 29:715–25. doi: 10.1038/jcbfm.2008.164, PMID: 19142198

[21] Ji ZJ Shi Y Li X Hou R Yang Y Liu ZQ. Neuroprotective effect of Taohong Siwu decoction on cerebral ischemia/reperfusion injury via Mitophagy-NLRP3 Inflammasome pathway. Front Pharmacol. (2022) 13:910217. doi: 10.3389/fphar.2022.910217, PMID: 35754465 PMC9213799

[22] Bederson JB Pitts LH Tsuji M Nishimura MC Davis RL Bartkowski H. Rat middle cerebral artery occlusion: evaluation of the model and development of a neurologic examination. Stroke. (1986) 17:472–6. doi: 10.1161/01.STR.17.3.472, PMID: 3715945

[23] Engelhorn T Goerike S Doerfler A Okorn C Forsting M Heusch G. The angiotensin II type 1-receptor blocker candesartan increases cerebral blood flow, reduces infarct size, and improves neurologic outcome after transient cerebral ischemia in rats. J Cereb Blood Flow Metab. (2004) 24:467–74. doi: 10.1097/00004647-200404000-00012, PMID: 15087716

[24] Choudhri TF Hoh BL Solomon RA Connolly ES Jr Pinsky DJ. Use of a spectrophotometric hemoglobin assay to objectively quantify intracerebral hemorrhage in mice. Stroke. (1997) 28:2296–302. doi: 10.1161/01.STR.28.11.2296, PMID: 9368579

[25] Yang F Cui Y Zhao Y Jiao H. Bibliometric analysis: research trends and performances of stroke on acupuncture. J Pain Res. (2024) 17:1837–51. doi: 10.2147/JPR.S449619, PMID: 38799275 PMC11128237

[26] Zhang ZH Zhang XC Ni GX. Thrombolysis combined with acupuncture therapy for acute cerebral infarction: a meta-analysis of randomized controlled trials. Zhen Ci Yan Jiu. (2021) 46:431–8. doi: 10.13702/j.1000-0607.200559, PMID: 34085469

[27] Zhang Z Lu T Li S Zhao R Li H Zhang X. Acupuncture extended the thrombolysis window by suppressing blood-brain barrier disruption and regulating autophagy-apoptosis balance after ischemic stroke. Brain Sci. (2024) 14:399. doi: 10.3390/brainsci14040399, PMID: 38672048 PMC11048240

[28] Yang ZX Xie JH Liu DD. Xingnao Kaiqiao needling method for acute ischemic stroke: a meta-analysis of safety and efficacy. Neural Regen Res. (2017) 12:1308–14. doi: 10.4103/1673-5374.213551, PMID: 28966646 PMC5607826

[29] Kim SJ Ahn DG Syed GH Siddiqui A. The essential role of mitochondrial dynamics in antiviral immunity. Mitochondrion. (2018) 41:21–7. doi: 10.1016/j.mito.2017.11.007, PMID: 29246869 PMC5988924

[30] Li W Sun K Hu F Chen L Zhang X Wang F. Protective effects of natural compounds against oxidative stress in ischemic diseases and cancers via activating the Nrf2 signaling pathway: a mini review. J Biochem Mol Toxicol. (2021) 35:e22658. doi: 10.1002/jbt.22658, PMID: 33118292

[31] Shen L Gan Q Yang Y Reis C Zhang Z Xu S. Mitophagy in cerebral ischemia and ischemia/reperfusion injury. Front Aging Neurosci. (2021) 13:687246. doi: 10.3389/fnagi.2021.687246, PMID: 34168551 PMC8217453

[32] Indo HP Davidson M Yen HC Suenaga S Tomita K Nishii T. Evidence of ROS generation by mitochondria in cells with impaired electron transport chain and mitochondrial DNA damage. Mitochondrion. (2007) 7:106–18. doi: 10.1016/j.mito.2006.11.026, PMID: 17307400

[33] Onishi M Yamano K Sato M Matsuda N Okamoto K. Molecular mechanisms and physiological functions of mitophagy. EMBO J. (2021) 40:e104705. doi: 10.15252/embj.2020104705, PMID: 33438778 PMC7849173

[34] He L Zhou Q Huang Z Xu J Zhou H Lv D. PINK1/Parkin-mediated mitophagy promotes apelin-13-induced vascular smooth muscle cell proliferation by AMPKα and exacerbates atherosclerotic lesions. J Cell Physiol. (2019) 234:8668–82. doi: 10.1002/jcp.27527, PMID: 30456860

[35] Quinn PMJ Moreira PI Ambrósio AF Alves CH. PINK1/PARKIN signalling in neurodegeneration and neuroinflammation. Acta Neuropathol Commun. (2020) 8:189. doi: 10.1186/s40478-020-01062-w, PMID: 33168089 PMC7654589

[36] Harper JW Ordureau A Heo JM. Building and decoding ubiquitin chains for mitophagy. Nat Rev Mol Cell Biol. (2018) 19:93–108. doi: 10.1038/nrm.2017.129, PMID: 29358684

[37] Connelly EM Frankel KS Shaw GS. Parkin and mitochondrial signalling. Cell Signal. (2023) 106:110631. doi: 10.1016/j.cellsig.2023.110631 36803775

[38] Han R Liu Y Li S Li XJ Yang W. PINK1-PRKN mediated mitophagy: differences between in vitro and in vivo models. Autophagy. (2023) 19:1396–405. doi: 10.1080/15548627.2022.2139080, PMID: 36282767 PMC10240983

[39] Durcan TM Fon EA. The three 'P's of mitophagy: PARKIN, PINK1, and post-translational modifications. Genes Dev. (2015) 29:989–99. doi: 10.1101/gad.262758.115, PMID: 25995186 PMC4441056

[40] Yamano K Matsuda N Tanaka K. The ubiquitin signal and autophagy: an orchestrated dance leading to mitochondrial degradation. EMBO Rep. (2016) 17:300–16. doi: 10.15252/embr.201541486, PMID: 26882551 PMC4772979

[41] Eldeeb MA Esmaili M Hassan M Ragheb MA. The role of PTEN-L in modulating PINK1-Parkin-mediated Mitophagy. Neurotox Res. (2022) 40:1103–14. doi: 10.1007/s12640-022-00475-w, PMID: 35699891

[42] Zhang X Yan H Yuan Y Gao J Shen Z Cheng Y. Cerebral ischemia-reperfusion-induced autophagy protects against neuronal injury by mitochondrial clearance. Autophagy. (2013) 9:1321–33. doi: 10.4161/auto.25132, PMID: 23800795

[43] Duan WL Wang XJ Ma YP Sheng ZM Dong H Zhang LY. Therapeutic strategies targeting the NLRP3-mediated inflammatory response and pyroptosis in cerebral ischemia/reperfusion injury (review). Mol Med Rep. (2024) 29:46. doi: 10.3892/mmr.2024.13170, PMID: 38275110 PMC10835666

[44] Jin R Liu L Zhang S Nanda A Li G. Role of inflammation and its mediators in acute ischemic stroke. J Cardiovasc Transl Res. (2013) 6:834–51. doi: 10.1007/s12265-013-9508-6, PMID: 24006091 PMC3829610

[45] Wang L Ren W Wu Q Liu T Wei Y Ding J. NLRP3 Inflammasome activation: a therapeutic target for cerebral ischemia-reperfusion injury. Front Mol Neurosci. (2022) 15:847440. doi: 10.3389/fnmol.2022.847440, PMID: 35600078 PMC9122020

[46] Heneka MT McManus RM Latz E. Inflammasome signalling in brain function and neurodegenerative disease. Nat Rev Neurosci. (2018) 19:610–21. doi: 10.1038/s41583-018-0055-7, PMID: 30206330

[47] Zhou R Yazdi AS Menu P Tschopp J. A role for mitochondria in NLRP3 inflammasome activation. Nature. (2011) 469:221–5. doi: 10.1038/nature09663, PMID: 21124315

[48] Wang W Chang R Wang Y Hou L Wang Q. Mitophagy-dependent mitochondrial ROS mediates 2,5-hexanedione-induced NLRP3 inflammasome activation in BV2 microglia. Neurotoxicology. (2023) 99:50–8. doi: 10.1016/j.neuro.2023.09.008, PMID: 37722613

[49] Mangan MSJ Olhava EJ Roush WR Seidel HM Glick GD Latz E. Targeting the NLRP3 inflammasome in inflammatory diseases. Nat Rev Drug Discov. (2018) 17:688. doi: 10.1038/nrd.2018.149, PMID: 30116046

[50] Chen X Wang Y Yao N Lin Z. Immunoproteasome modulates NLRP3 inflammasome-mediated neuroinflammation under cerebral ischaemia and reperfusion conditions. J Cell Mol Med. (2022) 26:462–74. doi: 10.1111/jcmm.17104, PMID: 34866334 PMC8743645

[51] Franke M Bieber M Kraft P Weber ANR Stoll G Schuhmann MK. The NLRP3 inflammasome drives inflammation in ischemia/reperfusion injury after transient middle cerebral artery occlusion in mice. Brain Behav Immun. (2021) 92:221–31. doi: 10.1016/j.bbi.2020.12.009, PMID: 33307174

[52] Xu Q Zhao B Ye Y Li Y Zhang Y Xiong X. Relevant mediators involved in and therapies targeting the inflammatory response induced by activation of the NLRP3 inflammasome in ischemic stroke. J Neuroinflammation. (2021) 18:123. doi: 10.1186/s12974-021-02137-8, PMID: 34059091 PMC8166383

[53] Xiao Q Kang Z Liu C Tang B. Panax notoginseng Saponins attenuate cerebral ischemia-reperfusion injury via Mitophagy-induced inhibition of NLRP3 Inflammasome in rats. Front Biosci. (2022) 27:300. doi: 10.31083/j.fbl2711300, PMID: 36472098

[54] Cao S Shrestha S Li J Yu X Chen J Yan F. Melatonin-mediated mitophagy protects against early brain injury after subarachnoid hemorrhage through inhibition of NLRP3 inflammasome activation. Sci Rep. (2017) 7:2417. doi: 10.1038/s41598-017-02679-z, PMID: 28546552 PMC5445068

[55] Hacke W Kaste M Fieschi C von Kummer R Davalos A Meier D. Randomised double-blind placebo-controlled trial of thrombolytic therapy with intravenous alteplase in acute ischaemic stroke (ECASS II). Second European-Australasian acute stroke study investigators. Lancet. (1998) 352:1245–51. doi: 10.1016/S0140-6736(98)08020-9, PMID: 9788453

[56] Wang Y Maeda T You S Chen C Liu L Zhou Z. Patterns and clinical implications of hemorrhagic transformation after thrombolysis in acute ischemic stroke: results from the ENCHANTED study. Neurology. (2024) 103:e210020. doi: 10.1212/WNL.0000000000210020, PMID: 39541551

[57] Hong JM Kim DS Kim M. Hemorrhagic transformation after ischemic stroke: mechanisms and management. Front Neurol. (2021) 12:703258. doi: 10.3389/fneur.2021.703258, PMID: 34917010 PMC8669478

[58] Zhong K An X Kong Y Chen Z. Predictive model for the risk of hemorrhagic transformation after rt-PA intravenous thrombolysis in patients with acute ischemic stroke: a systematic review and meta-analysis. Clin Neurol Neurosurg. (2024) 239:108225. doi: 10.1016/j.clineuro.2024.108225, PMID: 38479035

[59] Chen N Gao JD Zhao HS Liu SH Zhou YB Liu YS. Stratifying by blood glucose levels to predict hemorrhagic transformation risk post-Rt-PA in acute ischemic stroke. Clin Interv Aging. (2024) 19:1807–18. doi: 10.2147/CIA.S482060, PMID: 39525875 PMC11550918

[60] Ye Y Zhang FT Wang XY Tong HX Zhu YT. Antithrombotic agents for tPA-induced cerebral hemorrhage: a systematic review and Meta-analysis of preclinical studies. J Am Heart Assoc. (2020) 9:e017876. doi: 10.1161/JAHA.120.017876, PMID: 33283576 PMC7955384

[61] Gao F Du W Guo C Geng P Liu W Jin X. α7nACh receptor, a promising target to reduce BBB damage by regulating inflammation and autophagy after ischemic stroke. Biomed Pharmacother. (2024) 179:117337. doi: 10.1016/j.biopha.2024.117337, PMID: 39191022

[62] Jia W Lu R Martin TA Jiang WG. The role of claudin-5 in blood-brain barrier (BBB) and brain metastases (review). Mol Med Rep. (2014) 9:779–85. doi: 10.3892/mmr.2013.1875, PMID: 24366267

[63] Chen H Guan B Chen X Chen X Li C Qiu J. Baicalin attenuates blood-brain barrier disruption and hemorrhagic transformation and improves neurological outcome in ischemic stroke rats with delayed t-PA treatment: involvement of ONOO(−)-MMP-9 pathway. Transl Stroke Res. (2018) 9:515–29. doi: 10.1007/s12975-017-0598-3, PMID: 29275501

[64] He Q Li Z Wang Y Hou Y Li L Zhao J. Resveratrol alleviates cerebral ischemia/reperfusion injury in rats by inhibiting NLRP3 inflammasome activation through Sirt1-dependent autophagy induction. Int Immunopharmacol. (2017) 50:208–15. doi: 10.1016/j.intimp.2017.06.029, PMID: 28683365

[65] Palomino-Antolin A Narros-Fernández P Farré-Alins V Sevilla-Montero J Decouty-Pérez C Lopez-Rodriguez AB. Time-dependent dual effect of NLRP3 inflammasome in brain ischaemia. Br J Pharmacol. (2022) 179:1395–410. doi: 10.1111/bph.15732, PMID: 34773639

[66] Cheng X Ren Z Jia H Wang G. METTL3 mediates microglial activation and blood-brain barrier permeability in cerebral ischemic stroke by regulating NLRP3 Inflammasomes through m6A methylation modification. Neurotox Res. (2024) 42:15. doi: 10.1007/s12640-024-00687-2 38349604

[67] Yang F Wang Z Wei X Han H Meng X Zhang Y. NLRP3 deficiency ameliorates neurovascular damage in experimental ischemic stroke. J Cereb Blood Flow Metab. (2014) 34:660–7. doi: 10.1038/jcbfm.2013.242, PMID: 24424382 PMC3982086

[68] Ye Q Zhai F Chao B Cao L Xu Y Zhang P. Rates of intravenous thrombolysis and endovascular therapy for acute ischaemic stroke in China between 2019 and 2020. Lancet Reg Health West Pac. (2022) 21:100406. doi: 10.1016/j.lanwpc.2022.100406, PMID: 35243459 PMC8873940

[69] Fulton RL Lees KR Bluhmki E Biegert G Albers GW Davis SM. Selection for delayed intravenous alteplase treatment based on a prognostic score. Int J Stroke. (2015) 10:90–4. doi: 10.1111/j.1747-4949.2012.00943.x, PMID: 23294942

[70] Liang CX Xiao LY Gan JY Shi XX Wang XX Liu Y. Effects of acupuncture on hemorrhagic transformation and motor function in stroke patients after intravenous thrombolysis with rt-PA: a prospective cohort study. Zhongguo Zhen Jiu. (2023) 43:733–8. doi: 10.13703/j.0255-2930.20221206-0002, PMID: 37429649

