# Supplementary material for: Acupuncture alleviates hemorrhagic transformation after delayed rt-PA treatment for acute ischemic stroke by regulating the mitophagy-NLRP3 inflammasome pathway
Source: Front Neurol. 2025 Apr 7;16:1533092. doi: 10.3389/fneur.2025.1533092 (PMC12009825; doi:10.3389/fneur.2025.1533092)
Supplement: Supplementary file 1 [file Data_Sheet_1.pdf]

Supplementary Material

1 Supplementary Figures

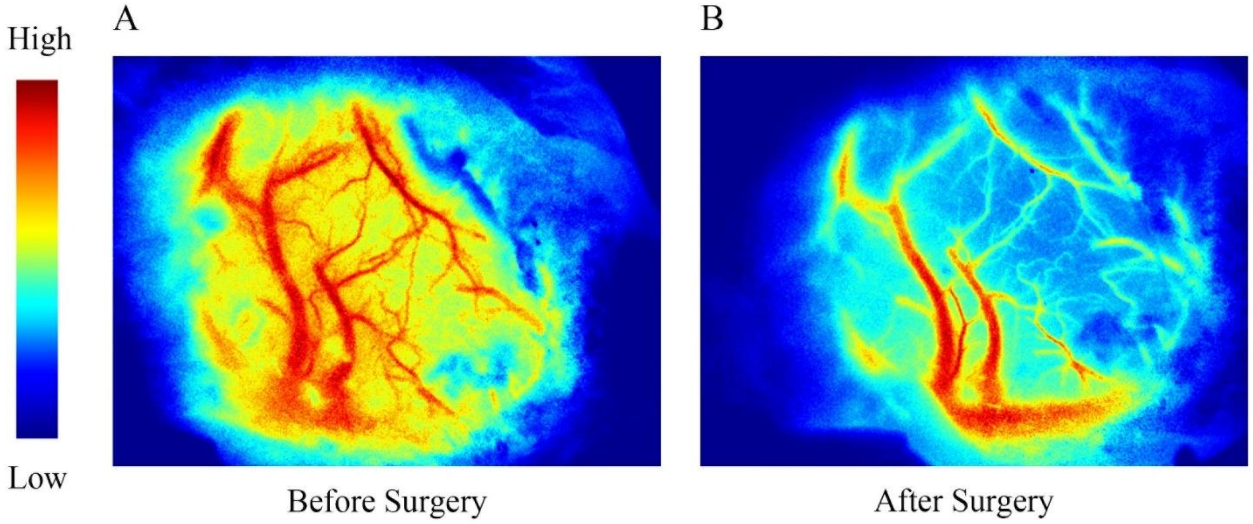

Figure 1. Cerebral blood flow was monitored by Laser speckle imaging during the surgery of embolic stroke model. A, B. Representative images of rCBF. Compared to the rCBF before surgery, the reduction of 50% or more in rCBF was considered as successful induction of ischemia.
